# Supplementary material for: Polygenic risk-tailored screening for prostate cancer: A benefit–harm and cost-effectiveness modelling study
Source: PLoS Med. 2019 Dec 20;16(12):e1002998. doi: 10.1371/journal.pmed.1002998 (PMC6924639; doi:10.1371/journal.pmed.1002998)
Supplement: S1 Appendix — (DOCX) [file pmed.1002998.s002.docx]

**Supplement 1:**

**Polygenic risk-tailored screening for prostate cancer: A benefit-harm and cost-effectiveness modelling study**

**Table of Contents**

[Supplementary Methods 3](#_Toc24040632)

[Table A: Costing data 4](#_Toc24040633)

[Costing descriptions 6](#_Toc24040634)

[Polygenic screening 6](#_Toc24040635)

[Treatment modalities 6](#_Toc24040636)

[Assessing suspected prostate cancer (PSA ≥ 3ng/ml) - biopsy 6](#_Toc24040637)

[Assessing suspected prostate cancer – if biopsy positive 6](#_Toc24040638)

[Active surveillance 7](#_Toc24040639)

[Follow-up after radical prostatectomy 7](#_Toc24040640)

[Follow-up after radical radiotherapy 7](#_Toc24040641)

[Complications after radical prostatectomy 8](#_Toc24040642)

[Complications after radiation therapy 8](#_Toc24040643)

[Resource use 9](#_Toc24040644)

[Biopsies 9](#_Toc24040645)

[PSA tests 9](#_Toc24040646)

[Treatments 9](#_Toc24040647)

[Utility estimates 9](#_Toc24040648)

[Overdiagnosis 10](#_Toc24040649)

[Model structure 10](#_Toc24040650)

[Figure A: Baseline model structure 10](#_Toc24040651)

[Life table 11](#_Toc24040652)

[Table B: Incidence and mortality estimates of prostate cancer in England, 2013-2016 11](#_Toc24040653)

[Absolute risk 12](#_Toc24040654)

[Figure B: 10-year absolute risk of developing prostate cancer at different ages of men in England, 2013-2016 12](#_Toc24040655)

[Figure C: Percentage of cases by percentage of the population above the risk threshold 13](#_Toc24040656)

[Figure D: 10-year absolute risk of prostate cancer in men aged 55 by percentile of the polygenic risk distribution 13](#_Toc24040657)

[Supplementary Results 14](#_Toc24040658)

[Table C: Outcomes of precision screening (starting from the age of 55) and age-based screening for prostate cancer as compared to no screening per 10,000 men screened (based on 10,000 simulations) 15](#_Toc24040659)

[Figure E: Percentage eligible for screening at different ages by 10-year absolute risk of prostate cancer 17](#_Toc24040660)

[Figure F: Ratio of overdiagnosed cases to prostate cancer deaths prevented with precision screening from age 55 compared with no screening 18](#_Toc24040661)

[Figure G: Ratio of overdiagnosed cases to prostate cancer deaths not averted with precision screening from age 55 compared with age-based screening 19](#_Toc24040662)

[Figure H: Net monetary benefits of no screening, age-based and precision screening from the age of 55 willingness-to-pay thresholds of £20,000 (A) and £30,000 (B) per QALY gained per 10,000 men 20](#_Toc24040663)

[Figure I: Cost-effectiveness planes of incremental cost vs. incremental QALYs of precision screening from age 55 compared to no screening, for 10-year absolute risk threshold between 2% and 10% (based on 10,000 simulations) 21](#_Toc24040664)

[Figure J: Cost-effectiveness acceptability curves of precision (from age 55) and age-based screening strategies at willingness-to-pay thresholds up to £100,000 per QALY 22](#_Toc24040665)

[Figure K: Cost-effectiveness acceptability frontier of precision screening strategies (from age 55) at willingness-to-pay thresholds up to £100,000 per QALY 23](#_Toc24040666)

[Figure L: Incidence of prostate cancer in the screened and unscreened cohorts of men in England, 2013-2016 24](#_Toc24040667)

[Figure M: Overdiagnosed cases and prostate cancer deaths prevented per 10,000 men screened assuming overdiagnosis varies by polygenic risk for precision screening from age 55 compared with (A) no screening and (B) age-based screening 25](#_Toc24040668)

[Figure N: Incremental cost-effectiveness ratios of precision screening from age 55 vs no screening by variances of the risk distribution of 0.60, 0.68, and 0.78 26](#_Toc24040669)

[Figure O: Incremental cost-effectiveness ratios of precision screening vs no screening at different levels of uptake and compliance with screening recommendations 27](#_Toc24040670)

[Figure P: Incremental cost-effectiveness ratios of precision screening vs no screening varying the cost of polygenic testing 28](#_Toc24040671)

[Table D: Outcomes per 10,000 men of age-based and precision screening compared with no screening from age 45 29](#_Toc24040672)

[Table E: Outcomes per 10,000 men of age-based and precision screening compared with no screening from age 50 30](#_Toc24040673)

[Table F: Outcomes per 10,000 men of age-based and precision screening compared with no screening from age 60 31](#_Toc24040674)

[Appendix references 32](#_Toc24040675)

**List of investigators:** Tom Callender^1^, Mark Emberton^2^, Steve Morris^1^, Ros Eeles^3^, Zsofia Kote-Jarai^3^, Paul DP Pharoah^4^, Nora Pashayan^1^

^1^ Department of Applied Health Research, Institute of Epidemiology & Health Care, University College London, London, UK.

^2^ Faculty of Medical Sciences, School of Life & Medical Sciences, University College London, London, UK.

^3^ The Institute of Cancer Research, London, UK.

^4^ Departments of Oncology, and Public Health and Primary Care, Strangeways Research Laboratory, University of Cambridge, Cambridge, UK

# Supplementary Methods

# Table A: Costing data

| **Resource** | **Description** | **Cost (£)** | **Source** |
| --- | --- | --- | --- |
| GP appointment |  | 36 | [1] |
| Polygenic risk assessment | Based on enquiries into the costs incurred by the PRACTICAL consortium. | 25 |  |
| Prostate specific antigen (PSA) test | £3 for phlebotomy (DAPS08), £1 for biochemistry (DAPS04) and the cost of phlebotomy. | 11.26 | [1,2] |
| Urology appointment | Average cost across both consultant and non-consultant-led first and subsequent outpatient appointments (service code 101). | 105.19 | [2] |
| Clinical oncology appointment | Average cost across both consultant and non-consultant-led first and subsequent outpatient appointments (service code 800). | 126.60 | [2] |
| Multidisciplinary team meeting | Cancer MDT (CMDT_OTH) | 107 | [2] |
| Prostate biopsy | £254 for a transrectal ultrasound guided biopsy (LB76Z) and £275 for a transperineal template biopsy (LB77Z). An estimated 88% of prostate biopsies are transrectal and 12% transperineal. | 256.52 | [2,3] |
| Hospital admission for biopsy complication (sepsis) | Weighted average of the HRG for sepsis without interventions (WJ06G/H/J).[2] | 1859.38 | [2] |
| Multiparametric MRI scan | T2, diffusion-weighted scan, as estimated by NICE. | 316 | [4] |
| Isotope bone scan | RN15A, ‘Nuclear Bone Scan of two or three phases, 19 years and over’.[2] | 242 | [2] |
| Radical prostatectomy | One appointment with an Urologist prior to surgery (£105.19).  Major Robotic, Prostate or Bladder Neck Procedures (LB69Z)[2] is £7284.16; Major laparoscopic, prostate or bladder neck procedures (LB22Z)[2] £5807.84; weighted average of major open, prostate or bladder neck procedures (LB21A/B)[2] £5199.30.  In 2016, 9.68% of radical prostatectomies were open, 11.06% laparoscopic, and 79.26% robotically assisted. These percentages were used to weight the average cost of radical prostatectomy per patient, which comes to £6919.06. | 7024.25 | [2,5] |
| Emergency readmission after radical prostatectomy | Average unit cost for a non-elective inpatient stay. | 3058.14 | [2] |
| Radical radiotherapy | One appointment with a clinical oncologist (£126.60). Preparation for Intensity Modulated Radiation Therapy (IMRT) (SC40Z)[2] costs £985. IMRT is the current recommended method for radical therapy.  Outpatient delivery of a fraction of treatment on a megavoltage machine[2] has a cost of £105 (SC22Z). There are two recommended regimens: 74-48Gy in 37-39 fractions or 60Gy in 20 fractions. In 2013/14, 58% received 37 fractions, 17% received 20 fractions. From this, it was assumed that approximately 1/3 of patients received 20 fractions, and the remainder 37, coming to a total per patient of £3290. | 4401.60 | [2,6–8] |
| GI complications of radiotherapy | Costs derived from the NICE (2014) based on Hummel et al. (2010), comprising weighted costs associated with investigation and management of GI complications. NICE estimate the one-off costs at £1611.46 in 2011/12, which have been inflated by 1.05 to 2015/16 prices. | 1694.17 | [6] |
| Brachytherapy | One appointment with a clinical oncologist (£126.60). Preparation for interstitial brachytherapy[2] is estimated to cost £730 (SC55Z), with delivery of a fraction of interstitial radiotherapy[2] costing £670 (SC28Z) | 1526.60 | [2] |
| Chemotherapy | Chemotherapy is used in metastatic disease. The most common treatment regimen is with docetaxel.  Administration of chemotherapy is assumed to involve two outpatient appointments with a clinical oncologist (£253.20). The cost of administration is a combination of the cost of parenteral chemotherapy at the first attendance (£253) (SB13Z), followed by the cost of subsequent rounds of chemotherapy (SB15Z) (£361), both as daycase procedures. Up to 10 cycles of docetaxel are recommended, however an average of 5 has been assumed as the proportion completing a full course is unknown. Docetaxel 75mg/m^2^ itself is estimated by NICE to cost £1023, based on a dose of 140mg. | 7426.20 | [2,6,9] |
| Androgen deprivation therapy | All individuals having hormone therapy are assumed to have a yearly urological appointment (£105.19). In 2014, NICE stated the costs of androgen deprivation therapies as follows: bicaultamide (£57 p.a.), flutamide (£491 p.a.) and leuprorelin (£903 p.a.). The average per patient per annum is £483, which has then been inflated by 1.022 to 2015/16 prices to £493.63. | 598.82 | [1,4] |
| Urinary incontinence | Derived from Mowatt et al. (2013) cited in NICE (2014). | 263.60 | [6] |
| Erectile dysfunction | Average unit cost for the treatment of erectile dysfunction (LB43Z) | 145 | [2] |
| Death from prostate cancer | The cost to the health care system in the last 12 months of life (£6687, 95% credible interval £535 to £20,257) has been inflated by 1.022 from 2013/14 to 2015/16 prices. | 6837 | [10] |
| Abbreviations: HCHS, Hospital and Community Health Services Index. GI, gastrointestinal. | | | |

## Costing descriptions

Average costs for each treatment modality were calculated using the NICE prostate cancer pathway and NICE prostate cancer guideline, incorporating treatment-related complications.[3,6,11,12] Where specific costings have been provided by NICE in their costing statement of the 2014 revision of the prostate cancer guidelines, these costs have been used over NHS Reference Costs. The cost estimated by NICE for the conduct of a diffusion-weighted dynamic contrast-enhanced multiparametric MRI (mpMRI) to guide transrectal ultrasound biopsy is £316.[6] Note that this value is higher than that the 2015/16 NHS Reference Cost for a “MRI scan of one area, with pre- and post-contrast” (RD03Z), which was £202.70.[2] This estimate was considered by the NICE guideline development group to be an underestimate of the true cost of mpMRI; consequently, the value used by NICE has been adopted.

## Polygenic screening

The cost of genetic screening has been estimated from personal discussion of the costs currently charged to NHS hospitals for prostate cancer genome-wide association studies. Genetic counselling is not assumed to be necessary as the composite risk score does not have specific familial implications. The precise format of polygenic screening (e.g. home testing swab-based kits) as well as the method by which results will be conveyed is not estimated.

## Treatment modalities

Resource use and ensuing costs have been estimated based on the NICE prostate cancer pathway[11] and NICE prostate cancer guideline CG175[6]:

| Assessing suspected prostate cancer (PSA ≥ 3ng/ml) - biopsy | | | |
| --- | --- | --- | --- |
| Stage of pathway | Description | Items | Cost (£) |
| Information and support for decision-making | 1. Urological appointment | 1 | 105.19 |
| Diagnosis & Staging | 1. Prostate biopsy (including assumption that 1.4% will be admitted to hospital post-biopsy)[13] | 1 | 283 |
|  | | | |
| *Total cost per patient* | | | 388.19 |

Those with a raised PSA (≥ 3ng/ml) are assumed to have one appointment with an urologist to discuss process of biopsy. The proportion invited back with a positive result are accounted for within the costs as ‘assessing suspected prostate cancer – if biopsy positive’ (see below). Individuals who have a raised PSA (≥ 3ng/ml) but refused biopsy were assumed to have had both a urological appointment, giving a total for these men of £105.19.

| Assessing suspected prostate cancer – if biopsy positive | | | |
| --- | --- | --- | --- |
| Stage of pathway | Description | Items | Cost (£) |
| Diagnosis & Staging | 1. Multiparametric MRI (other than to those who not having radical therapy) | 1 | 316 |
|  | 1. Isotope bone scan | 1 | 242 |
|  | 1. Urological MDT | 1 | 107 |
|  | 1. Urological appointment | 1 | 105.19 |
|  | | | |
| *Total cost per patient* | | | 770.19 |

The proportion having mpMRI after a negative biopsy to decide upon further having another biopsy is unknown and is assumed to be low. The proportion having repeat biopsies is not known and has not been included. Individuals who are diagnosed with stage three or four prostate cancer were assumed to have a staging MRI and isotope bone scan.

Resource use related to watchful waiting and active surveillance, radiotherapy, chemotherapy and radical prostatectomy was allocated using the NICE prostate cancer pathway.[11]

| Active surveillance | | | |
| --- | --- | --- | --- |
| Year(s) | Description | Items | Cost (£) |
| 1 | PSA test | 3 | 33.78 |
|  | Urological appointment (digital rectal examination [DRE]) | 2 | 210.38 |
|  | Prostate biopsy | 1 | 256.52 |
| 2-4 | PSA test | 3 | 101.34 |
|  | Urological appointment (DRE) | 2 | 631.14 |
| 5 onwards | PSA test | 2 per year for 5 years | 112.60 |
|  | Urological appointment (DRE) | 1 per year for 5 years | 525.95 |
|  | | | |
| *Total cost per patient* | | | 1871.71 |

To create an average cost for an individual undertaking a strategy of active surveillance, a timeframe of 10-years has been taken, with an assumption that 45% will have active surveillance for this period of time, based on the ProtecT trial,[14] and the remainder will go on to have radical therapy in the form of either radical prostatectomy or radical radiotherapy. It has been assumed that the proportions receiving either form of radical therapy mirror the broader proportions of individuals receiving the respective therapies (16% radical prostatectomy and 30% radical radiotherapy) in the first year of diagnosis.[15]

Watchful waiting is usually considered palliative in intent, with the aim to provide symptomatic management as necessary.[12,16] Each urological MDT may have different protocols for the management of those who have opted for a strategy of watchful waiting, however NICE recommends at least once yearly PSA testing.[11] The proportion of individuals having watchful waiting, and the time for which they might have this, is unknown. The ensuing costs from once yearly PSA testing and a GP appointment are negligible; as a result, the average costs of watchful waiting have not been estimated.

| Follow-up after radical prostatectomy | | | |
| --- | --- | --- | --- |
| Year | Description | Items | Cost (£) |
| 1 | PSA test | 4 | 45.04 |
|  | Urological appointment | 4 | 420.76 |
| 2 | PSA test | 2 | 22.52 |
|  | Urological appointment | 2 | 210.38 |
| 3 onwards | PSA test | 1 per year for 3 years | 33.78 |
|  | GP appointment | 1 per year for 3 years | 108 |
|  | | | |
| *Total cost per patient* | | | 840.48 |
|  | | | |
| Follow-up after radical radiotherapy | | | |
| Year | Description | Items | Cost (£) |
| 1-2 | PSA test | 2 per year | 45.04 |
|  | Clinical oncology appointment | 2 per year | 506.40 |
| 3 onwards | PSA test | 1 per year for 3 years | 33.78 |
|  | GP appointment | 1 per year for 3 years | 108 |
|  | | | |
| *Total cost per patient* | | | 693.22 |

NICE guidance does not provide a precise protocol as to follow-up, consequently resource-use during follow-up after radical prostatectomy has been estimated based on the protocol of the ProtecT trial.[17] After at least two years, follow-up can be provided in primary care, or via telephone or secure electronic communication.[11] We have assumed that this will consist of a yearly GP appointment, and have estimated costs over a five-year timeframe.

| Complications after radical prostatectomy | |  |
| --- | --- | --- |
| *Complication* | *Description* | *Source* |
| Erectile dysfunction | 65% based on Fenton et al. (2018) meta-analysis of 3 good-quality RCTs. | [12] |
| Urinary incontinence | 22.9% of those treated with radical prostatectomy, based on Fenton et al. (2018) meta-analysis of 3 good-quality RCTs. | [12] |
| Emergency readmission | 5% had an emergency readmission within 90 days of RP. Assumed average cost of a non-elective inpatient stay. | [3] |
|  | | |
| Complications after radiation therapy | |  |
| *Complication* | *Description* | *Source* |
| Urinary incontinence | No clear association seen across 2 RCTs; substantial variation in outcomes and measurement in 6 cohort studies with estimates ranging from 2.4% to 16.9%.  Donovan et al. (2016) estimate of 3.5% from ProtecT used as most recent English data from a good-quality RCT. | [12,18] |
| Erectile dysfunction | 65% based on a meta-analysis of 3 cohort studies. | [12] |
| Severe gastrointestinal complication | 11% within 2 years of radical radiotherapy. | [3] |

The cost of complications after radical treatment are estimated over a ten-year timeframe, with the exception of emergency readmissions which are treated as a one-off, as are the costs associated with severe gastrointestinal complications from radiotherapy.

The average cost for a radical prostatectomy was therefore estimated as a combination of the cost of surgery (£7024) and relevant consultant appointments combined with the cost of follow-up (£841). An additional five percent were assumed to have an emergency readmission, 65% erectile dysfunction and 23% urinary incontinence (see Table).[12]

The average cost for radical radiotherapy was therefore estimated as a combination of the cost of the radiotherapy planning and the radiotherapy itself (£4402) combined with the cost of follow-up (£693). Costs due to erectile dysfunction, urinary incontinence, and severe gastrointestinal complications are applied to 65%, 3.5%, and 11% of those receiving radical radiotherapy, respectively (see Table).[3,12,18]

Costs arising from death from causes other than prostate cancer have not been estimated.

# Resource use

## Biopsies

Resource use for biopsy was estimated as follows:

- In non-screened cohort, the number of cases of cancer diagnosed was divided by 0.35. This was calculated from the number of cancers detected per biopsy performed in a population of English men clinically-suspected to have prostate cancer (e.g. PSA ≥ 3ng/ml) in the PROMIS trial.[19] From this the number of biopsies was estimated. Total costs accrued by biopsies consisted both of the costs attributed to the biopsies performed, and costs attributed to those who had a PSA ≥ 3ng / ml but chose not to have a biopsy (estimated from ProtecT).[17]
- In the screened cohort, the number of cancers was divided by 0.24, which reflects the proportion of screen-detected cancers per biopsy in ERSPC.[20]
- The uncertainty in the proportion of cancers detected per biopsy was modelled using the normal distribution with a standard deviation of 0.05.

## PSA tests

In the non-screening arm, the number of PSA tests occurring in the community for clinically suspected cancer was estimated as the number of biopsies performed multiplied by 20%. The uncertainty in this figure was modelled in probabilistic sensitivity analyses using the normal distribution and a standard deviation of 0.05.

## Treatments

Treatment for prostate cancer was divided into active surveillance, radical therapies (radical prostatectomy, radical radiotherapy, and brachytherapy), androgen deprivation therapy, and chemotherapy. Public Health England’s National Cancer Registration and Analysis Service (NCRAS) provides the proportions receiving different combinations of radical prostatectomy, radical radiotherapy, chemotherapy, and other treatments, in the first 12-15 months from diagnosis between 2013-2015.[15] These data are sourced from three mandatory datasets: the Systemic Anti-Cancer Therapy registry, the Radiotherapy Dataset (RTDS), and Hospital Episode Statistics.[15] Data on the proportions receiving active surveillance, brachytherapy, and androgen deprivation therapy are not captured by this dataset.

Active surveillance is a recommended therapeutic option for individuals with low-risk localised disease.[6] Consequently, the proportion receiving active surveillance was assumed to be equal to the proportion of patients diagnosed in 2016 with low-risk localised cancer who did not have radical therapy, as recorded in the National Prostate Cancer Audit (NPCA), inflated by 17% to account for the findings of ProtecT and the use of active surveillance amongst individuals with other localised cancers.[3] The proportion receiving brachytherapy was obtained from RTDS through the NPCA whilst the proportion of individuals receiving androgen deprivation therapy was obtained from the NICE prostate cancer guidelines costing statement and inflated amongst those with stage four disease to account for its use in conjunction with chemotherapy for those with hormone-sensitive metastatic cancer.[3,4] The proportions receiving these three treatment modalities – active surveillance, brachytherapy, and androgen deprivation therapy – were not varied by age due to an absence of relevant data.

# Utility estimates

We used the background age-specific EQ-5D-based utility estimates for the general English population, including both those with and without comorbidities, from the Health Survey for England.[21] From these utility estimates, an average yearly decrement in utility of 0.0040 (standard error of 0.0005) was derived using linear regression.[21] This yearly decrement was applied to both those with a diagnosis of prostate cancer and those without such a diagnosis.

Due to the heterogeneity of available estimates in the literature, and based on the method of Pharoah and colleagues,[22] we developed a single score for those with prostate cancer undergoing radical prostatectomy, radical radiotherapy and active surveillance. We used the utility value of 0.78 of Hummel and colleagues to represent the first year after radical therapy. In line with the findings of Downing,[23] and following precedent,[24,25] it is assumed that the longer term average score will return towards baseline over a longer period. An estimate of 0.95 from year two post-treatment onwards was used.[24,25] As the ten-year survival rate of prostate cancer is 83.8%, an average value of over ten years was used,[26] leading to a 10-year average score of 0.93 for those having radical therapy. For those pursuing a strategy of active surveillance, a score of 0.97 was used by Heijnsdijk and colleagues over a 9 year period, which correlates with recent evidence that those undertaking this course of action have scores comparable to the background population, and higher than those having radical treatment.[27–29] Approximately 55% of individuals initially managed with active surveillance in England are likely to have radical therapy within ten years. Applying these proportions, the average ten-year score for active surveillance was calculated as 0.95, the average of 0.97 and 0.93. The final utility value for individuals with prostate cancer was 0.93 relative to the background population utility. The utility estimate for those with prostate cancer was adjusted by the age-specific population average utility.

There are several longitudinal studies of health-related quality of life in individuals with prostate cancer. Korfage and colleagues found that utility scores, measured using the EQ-5D, remained relatively stable over five years in those having radical prostatectomy (0.89) or radiotherapy (0.81).[30] Downing and colleagues, also using EQ-5D, showed in a population-based study of 35,832 UK patients who have had prostate cancer for 18-42 months, that self-assessed health varied little between disease stage and was similar to, if not higher than, the background population.[23] This suggests that a score of 0.93 relative to the background population is likely to be representative, if possibly conservative in its estimate of the average health-related quality-of-life in an individual with prostate cancer over a 10 year period.

## Overdiagnosis

Model-based estimates of overdiagnosis with PSA screening range from 10% amongst those aged 50-54 to 31% between the ages of 65-69.[31] These estimates were regressed against age to derive the yearly proportion at risk of overdiagnosis of -0.62 + age x 0.014.

# Model structure

Prostate cancer can be categorised into four stages depending on the size of the tumour and its spread.[32] Individuals are expected to progress from early-stage to advanced-stage disease over time, however the transition rates between stages remain unclear and are likely to vary substantially between individuals, with some disease remaining dormant and other cases progressing relatively rapidly. In addition, the impact of polygenic risk on transition rates remains unknown. In view of this, a model that independently considers an individual’s progression through each stage of the disease was thought to introduce substantial uncertainty, whilst impacting model transparency. Consequently, a life-table approach was taken, and the model developed with high-quality aggregate population-level incidence and mortality data from the Office for National Statistics (ONS).[33,34]

# Figure A: Baseline model structure

*
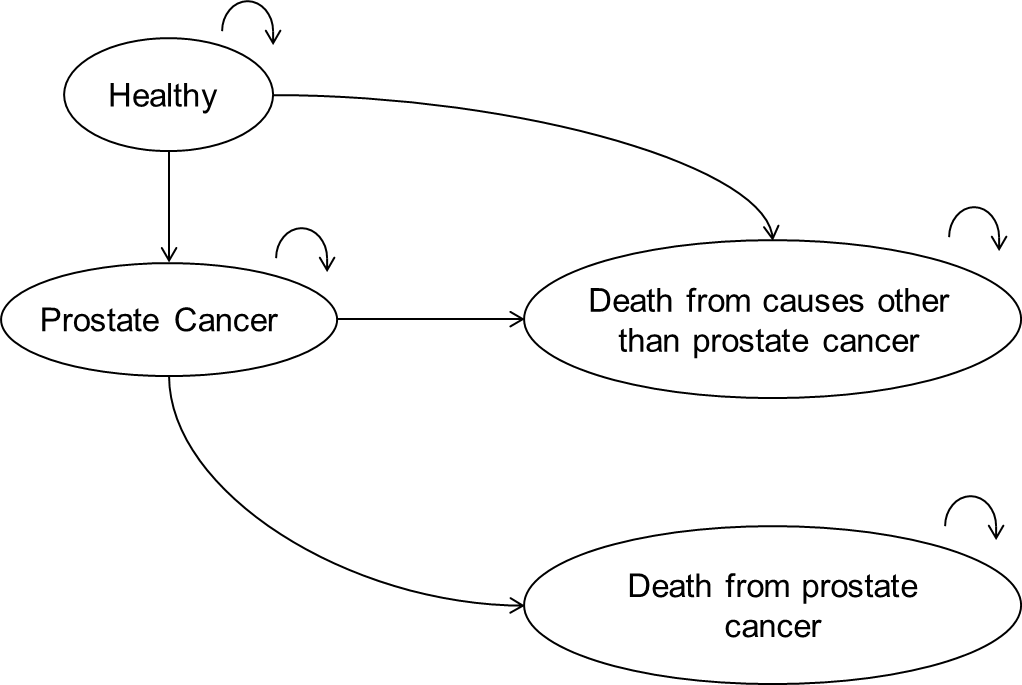
*

With each cycle of the model, healthy individuals have both an age-dependent risk of developing prostate cancer as well as an age-dependent risk of death from causes other than prostate cancer. Those with prostate cancer have an age-dependent yearly risk of death from both prostate cancer and death from other causes.

## Life table

A multistate life-table estimates the proportion of the population in a particular state at different ages.[35] As shown in S1 Figure 1, four states were modelled: healthy, which reflects those individuals who do not have prostate cancer and have not died, prostate cancer, death from prostate cancer, and death from causes other than prostate cancer. ONS provides incidence and mortality data from prostate cancer divided into five-yearly age groups.[25,33,34,36] The log of these figures was regressed against age to derive yearly values (S1 Table 2); these predicted values were used to populate the life-table.

# Table B: Incidence and mortality estimates of prostate cancer in England, 2013-2016

| **Age Group** | **Prostate Cancer Cases (n)** | **Deaths from Prostate Cancer (n)** | **Deaths from Other Causes (n)** | **Male Population (n)** | **Incidence of Prostate Cancer** | **Mortality from Prostate Cancer** | **Mortality from Other Causes** |
| --- | --- | --- | --- | --- | --- | --- | --- |
| 45-49 | 372 | 16 | 4,730 | 1,929,137 | 0.00019 | 0.000008 | 0.00245 |
| 50-54 | 1,264 | 50 | 6,698 | 1,856,908 | 0.00068 | 0.00003 | 0.00361 |
| 55-59 | 2,986 | 144 | 9,066 | 1,601,952 | 0.00186 | 0.00009 | 0.00566 |
| 60-64 | 4,922 | 340 | 13,069 | 1,431,345 | 0.00344 | 0.00024 | 0.00913 |
| 65-69 | 8,676 | 770 | 19,814 | 1,450,408 | 0.00598 | 0.00053 | 0.01366 |
| 70-74 | 7,747 | 1,094 | 23,938 | 1,064,547 | 0.00728 | 0.00103 | 0.02249 |
| 75-79 | 6,895 | 1,592 | 31,178 | 819,504 | 0.00841 | 0.00194 | 0.03804 |
| 80-84 | 4,018 | 2,056 | 38,184 | 566,378 | 0.00709 | 0.00363 | 0.06742 |
| 85-89 | 2,258 | 2,036 | 36,902 | 307,822 | 0.00734 | 0.00661 | 0.11988 |

The average number of incident prostate cancer cases, deaths from prostate cancer and deaths from other causes are shown in S1 Table 2 along with the average number of men in the population for each relevant five-year age group. These data were calculated from the relevant data from the Office for National Statistics for the years 2013 to 2016.[33,36–38]

In the presence of screening, cases of prostate cancer are diagnosed earlier than would have occurred otherwise.[39] The time by which a diagnosis is brought forward depending on the sojourn time, which has been estimated at 11.3 to 12.6 years for prostate cancer in the context of PSA screening.[31] Consequently, in the first few years after the cessation of screening, the incidence of prostate cancer will drop as some cases that might have presented clinically in the absence of a screening programme will have already been screen-detected at an earlier age. On the cessation of screening, the incidence in the screened groups will drop given the advance in diagnosis seen with the screening programme. The incidence in the screened cohorts was adjusted by 10%.

Additionally, the introduction of screening across a population is not a uniform event, such that the full impact of screening on incidence and mortality will not be immediately felt. A gradual change was therefore applied to the relative risk for prostate cancer mortality over the five years from the start of screening and from the cessation of screening, with five years an assumption as the true figure is unknown. For example, the relative risk of death from prostate cancer decreased from 1.0 to 0.79 over the same timeframe.

There is no screening programme for prostate cancer in England, although informal, and perhaps inadvertent, screening is likely to occur given that the ten-year risk of having a PSA test in English General Practice is 53% amongst men aged 65-69.[40] PSA is not specific to prostate cancer and is used in the investigation of certain urinary tract symptoms.[40] The proportion at risk of a PSA test rises with age, and it is unknown what proportions of these tests were done in the investigation of other conditions, in those with a clinical suspicion of cancer, and in those who are being screened for prostate cancer in the absence of symptoms.[40] Consequently, up to 10% of men in the no screening arm of the model were assumed to have had some form of screening by the age of 90, with the incidence in the no screening arm of the model was adjusted accordingly. The true proportion receiving these tests due to suspected prostate cancer is unknown.[40]

## Absolute risk

The variance of the polygenic risk distribution was estimated as 0.68 based on known prostate cancer susceptibility variants.[41,42]

The 10-year absolute risks of developing prostate cancer for yearly age groups from 50 to 79 was calculated using DevCan version 6.7.6 (US National Cancer Institute, USA) from the mean of the incidence of prostate cancer, mortality from prostate cancer and mortality from other causes between 2013 and 2016 as recorded by the Office for National Statistics (S1 Figure 2).[43]

We calculated the age-specific log relative risk of developing prostate cancer and from this and the polygenic risk distribution amongst cases, the relative risk of developing prostate cancer amongst those above and below the risk threshold (S1 Figure 3).

# Figure B: 10-year absolute risk of developing prostate cancer at different ages of men in England, 2013-2016

S1 Figure 3 shows the proportion of cases amongst those above the risk threshold, based on a risk distribution with a variance of 0.68. The percentile of the risk distribution is equivalent to 100 – the percentage above each percentage threshold. For example, 49% of cancers occur in men in the top 20^th^ percentile of the risk distribution. S1 Figure 4 shows the relationship between the 10-year absolute risk of developing prostate cancer for a man aged 55 at each percentile of the risk distribution.

# Figure C: Percentage of cases by percentage of the population above the risk threshold

# Figure D: 10-year absolute risk of prostate cancer in men aged 55 by percentile of the polygenic risk distribution

# Supplementary Results

# Table C: Outcomes of precision screening (starting from the age of 55) and age-based screening for prostate cancer as compared to no screening per 10,000 men screened (based on 10,000 simulations)

| **Screening strategy** | **ICER (£ / QALY)** | **Prostate cancer cases (n)** | **Overdiagnosed Cases (n)** | **Deaths from prostate cancer (n)** | **Total life-years (n)** | **QALYs with prostate cancer (n)** | **QALYs - healthy (n)** | **Total QALYs (n)** | **Biopsies (n)** | **Total Costs (£)** | **Cumulative percentage screened (%)** |
| --- | --- | --- | --- | --- | --- | --- | --- | --- | --- | --- | --- |
| No Screening | - | 1,200 | - | 430 | 134,547 | 4,087 | 100,030 | 104,117 | 3,454 | 6,636,010 |  |
| Age-based screening | 34,952 | 1,436 | 212 | 342 | 134,845 | 6,152 | 98,002 | 104,154 | 5,159 | 7,915,655 | 100 |
| *Difference with no screening* | *-* | *237* | *-* | *-88* | *298* | *2,065* | *-2,029* | *37* | *1,705* | *1,279,645* |  |
| 10-year AR of 2.0% | 30,297 | 1,389 | 189 | 348 | 134,823 | 5,885 | 98,276 | 104,161 | 4,909 | 7,967,737 | 75.4 |
| *Difference with no screening* | *-* | *189* | *-* | *-81* | *276* | *1,798* | *-1,754* | *44* | *1,455* | *1,331,727* |  |
| 10-year AR of 2.5% | 27,542 | 1,370 | 178 | 352 | 134,810 | 5,762 | 98,401 | 104,162 | 4,798 | 7,889,188 | 66.7 |
| *Difference with no screening* | *-* | *170* | *-* | *-78* | *263* | *1,675* | *-1,630* | *46* | *1,344* | *1,253,178* |  |
| 10-year AR of 3.0% | 25,290 | 1,352 | 166 | 356 | 134,796 | 5,638 | 98,526 | 104,163 | 4,688 | 7,811,666 | 58.9 |
| *Difference with no screening* | *-* | *152* | *-* | *-74* | *250* | *1,551* | *-1,505* | *46* | *1,234* | *1,175,656* |  |
| 10-year AR of 3.5% | 23,446 | 1,334 | 155 | 360 | 134,783 | 5,517 | 98,647 | 104,164 | 4,582 | 7,737,606 | 51.9 |
| *Difference with no screening* | *-* | *135* | *-* | *-70* | *236* | *1,430* | *-1,383* | *47* | *1,128* | *1,101,596* |  |
| 10-year AR of 4.0% | 21,924 | 1,319 | 144 | 364 | 134,769 | 5,402 | 98,762 | 104,164 | 4,483 | 7,668,183 | 45.8 |
| *Difference with no screening* | *-* | *119* | *-* | *-66* | *222* | *1,316* | *-1,269* | *47* | *1,029* | *1,032,173* |  |
| 10-year AR of 4.5% | 20,659 | 1,304 | 133 | 367 | 134,756 | 5,295 | 98,869 | 104,164 | 4,391 | 7,603,852 | 40.5 |
| *Difference with no screening* | *-* | *105* | *-* | *-62* | *209* | *1,208* | *-1,162* | *47* | *937* | *967,842* |  |
| 10-year AR of 5.0% | 19,598 | 1,291 | 124 | 371 | 134,743 | 5,196 | 98,968 | 104,163 | 4,307 | 7,544,664 | 35.9 |
| *Difference with no screening* | *-* | *92* | *-* | *-58* | *196* | *1,109* | *-1,063* | *46* | *853* | *908,654* |  |
| 10-year AR of 5.5% | 18,704 | 1,280 | 115 | 375 | 134,731 | 5,104 | 99,059 | 104,163 | 4,230 | 7,490,443 | 31.9 |
| *Difference with no screening* | *-* | *80* | *-* | *-55* | *184* | *1,017* | *-972* | *46* | *776* | *854,433* |  |
| 10-year AR of 6.0% | 17,947 | 1,269 | 106 | 378 | 134,720 | 5,020 | 99,142 | 104,162 | 4,160 | 7,440,903 | 28.4 |
| *Difference with no screening* | *-* | *70* | *-* | *-52* | *173* | *933* | *-888* | *45* | *706* | *804,893* |  |
| 10-year AR of 6.5% | 17,303 | 1,260 | 99 | 381 | 134,709 | 4,943 | 99,218 | 104,161 | 4,096 | 7,395,702 | 25.4 |
| *Difference with no screening* | *-* | *61* | *-* | *-49* | *163* | *856* | *-813* | *44* | *642* | *759,692* |  |
| 10-year AR of 7.0% | 16,755 | 1,252 | 92 | 384 | 134,699 | 4,873 | 99,287 | 104,160 | 4,038 | 7,354,488 | 22.7 |
| *Difference with no screening* | *-* | *52* | *-* | *-46* | *153* | *786* | *-743* | *43* | *584* | *718,477* |  |
| 10-year AR of 7.5% | 16,289 | 1,245 | 85 | 386 | 134,690 | 4,809 | 99,350 | 104,159 | 3,986 | 7,316,910 | 20.4 |
| *Difference with no screening* | *-* | *45* | *-* | *-43* | *144* | *722* | *-680* | *42* | *532* | *680,900* |  |
| 10-year AR of 8.0% | 15,894 | 1,238 | 79 | 389 | 134,682 | 4,750 | 99,407 | 104,158 | 3,939 | 7,282,637 | 18.4 |
| *Difference with no screening* | *-* | *39* | *-* | *-41* | *135* | *663* | *-623* | *41* | *485* | *646,627* |  |
| 10-year AR of 8.5% | 15,560 | 1,232 | 74 | 391 | 134,674 | 4,697 | 99,460 | 104,156 | 3,896 | 7,251,362 | 16.6 |
| *Difference with no screening* | *-* | *33* | *-* | *-38* | *127* | *610* | *-571* | *40* | *442* | *615,352* |  |
| 10-year AR of 9.0% | 15,281 | 1,227 | 69 | 393 | 134,666 | 4,648 | 99,507 | 104,155 | 3,857 | 7,222,799 | 15.0 |
| *Difference with no screening* | *-* | *28* | *-* | *-36* | *120* | *561* | *-523* | *38* | *403* | *586,789* |  |
| 10-year AR of 9.5% | 15,050 | 1,223 | 64 | 395 | 134,659 | 4,604 | 99,551 | 104,154 | 3,822 | 7,196,692 | 13.6 |
| *Difference with no screening* | *-* | *23* | *-* | *-34* | *113* | *517* | *-480* | *37* | *368* | *560,682* |  |
| 10-year AR of 10.0% | 14,862 | 1,219 | 60 | 397 | 134,653 | 4,563 | 99,590 | 104,153 | 3,790 | 7,172,805 | 12.3 |
| *Difference with no screening* | *-* | *19* | *-* | *-32* | *106* | *476* | *-440* | *36* | *336* | *536,795* |  |

Abbreviations: ICER, incremental cost-effectiveness ratio; QALYs, quality-adjusted life-years; AR, absolute risk.

# Figure E: Percentage eligible for screening at different ages by 10-year absolute risk of prostate cancer

Lines are labelled with the corresponding 10-year absolute risk threshold. For example, at the age of 55, 46% of men would be eligible for screening, reaching 87% at age 69. In order to preserve readability, selected risk-thresholds are presented.

# Figure F: Ratio of overdiagnosed cases to prostate cancer deaths prevented with precision screening from age 55 compared with no screening

Results are based on 10,000 simulations.

# Figure G: Ratio of overdiagnosed cases to prostate cancer deaths not averted with precision screening from age 55 compared with age-based screening

This represents the ratio of overdiagnoses avoided for every prostate cancer not averted with precision screening compared with age-based screening. Results are based on 10,000 simulations. By comparison with age-based screening, as the 10-year absolute risk threshold was increased from 2.0% to 10.0% in the precision screening cohort, the ratio of overdiagnosed cancers avoided for each prostate cancer death not prevented reduced from 3.7 to 2.7.

# Figure H: Net monetary benefits of no screening, age-based and precision screening from the age of 55 willingness-to-pay thresholds of £20,000 (A) and £30,000 (B) per QALY gained per 10,000 men

**A B**

Results based on 10,000 simulations.

# Figure I: Cost-effectiveness planes of incremental cost vs. incremental QALYs of precision screening from age 55 compared to no screening, for 10-year absolute risk threshold between 2% and 10% (based on 10,000 simulations)

**
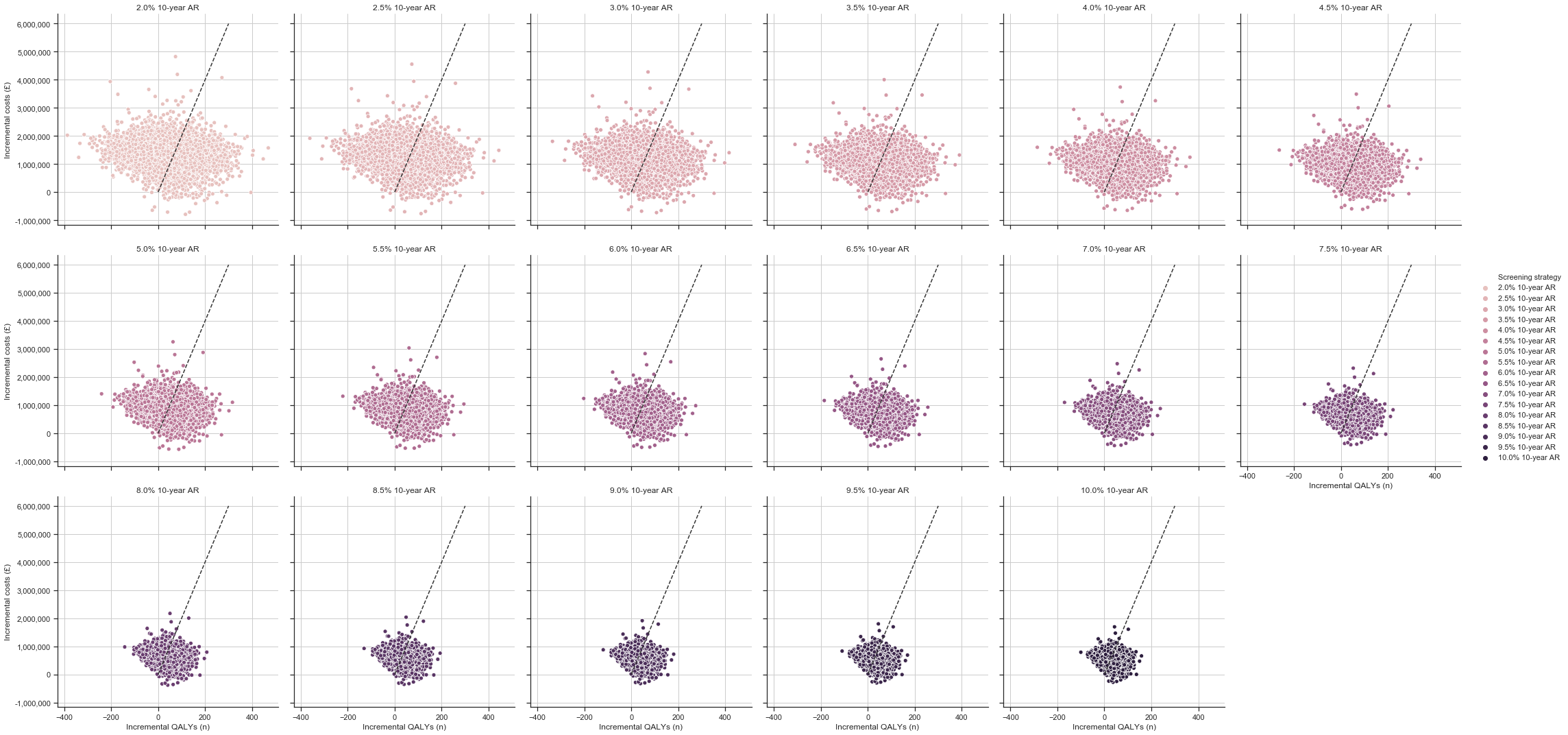
**

# Figure J: Cost-effectiveness acceptability curves of precision (from age 55) and age-based screening strategies at willingness-to-pay thresholds up to £100,000 per QALY

Cost-effectiveness acceptability curves for selected screening strategies vs no screening. The vertical dashed black and red lines highlight the willingness-to-pay thresholds of £20,000 and £30,000 per QALY, respectively, which represent the range considered cost-effective by the UK National Institute for Health and Care Excellence.[44] Results are based on 10,000 simulations.

# Figure K: Cost-effectiveness acceptability frontier of precision screening strategies (from age 55) at willingness-to-pay thresholds up to £100,000 per QALY

The cost-effectiveness acceptability frontier shows the precision screening strategy that offers the highest net benefit at each willingness-to-pay threshold, as well as the probability that the strategy is cost-effective. The vertical dashed black and red lines highlight the willingness-to-pay thresholds of £20,000 and £30,000 per QALY gained. At these points, precision screening at a 10-year absolute risk threshold of 10% and 8% had the highest net monetary benefit, respectively. Results are based on 10,000 simulations.

# Figure L: Incidence of prostate cancer in the screened and unscreened cohorts of men in England, 2013-2016

Incidence with age-based vs no screening. Results based on 10,000 simulations.

# Figure M: Overdiagnosed cases and prostate cancer deaths prevented per 10,000 men screened assuming overdiagnosis varies by polygenic risk for precision screening from age 55 compared with (A) no screening and (B) age-based screening

**A B**

Sensitivity analysis in which overdiagnosis is assumed to vary by polygenic risk. In this sensitivity analyses, overdiagnosis was multiplied by the reciprocal of the relative risk of prostate cancer for those above and below the risk threshold in order to model overdiagnosis under the assumption that it does indeed vary by polygenic risk. Results based on 10,000 simulations.

# Figure N: Incremental cost-effectiveness ratios of precision screening from age 55 vs no screening by variances of the risk distribution of 0.60, 0.68, and 0.78

In sensitivity analyses, the variance of the risk distribution was varied by the confidence intervals of the most recent estimates of the familial relative risk of prostate cancer explained by known risk loci (37%, 95% CI: 33% – 43%).^45^ The corresponding variances of the risk distribution are 0.68 (0.60 – 0.78). Results based on 10,000 simulations.

# Figure O: Incremental cost-effectiveness ratios of precision screening vs no screening at different levels of uptake and compliance with screening recommendations

Three parameters were varied: the uptake of polygenic stratification, the uptake of PSA screening in those eligible (above the risk threshold), and compliance with the recommendation to have or to forego PSA screening. In each scenario, only the parameter described was varied. For example, in the scenario modelling 75% compliance with the screening recommendation, uptake of both polygenic risk stratification and PSA screening in those above the risk threshold was assumed to be 100%. Results based on 10,000 simulations.

# Figure P: Incremental cost-effectiveness ratios of precision screening vs no screening varying the cost of polygenic testing

Results based on 10,000 simulations.

# Table D: Outcomes per 10,000 men of age-based and precision screening compared with no screening from age 45

| **Screening strategy** | **Prostate cancer cases (n)** | ***Difference with no screening*** | **Overdiagnosed cases (n)** | **Deaths from prostate cancer (n)** | ***Difference with no screening*** | **QALYs (n)** | ***Difference with no screening*** | **Costs (£)** | ***Difference with no screening*** | **ICER (£ / QALY gained)** |
| --- | --- | --- | --- | --- | --- | --- | --- | --- | --- | --- |
| No screening | 1,264 |  | - | 422 |  | 123,279 |  | 5,966,991 |  |  |
| Age-based screening | 1,512 | *248* | 229 | 336 | *-86* | 123,295 | *16* | 7,296,889 | *1,329,898* | 85,023 |
| Precision screening (10-year AR) |  |  |  |  |  |  |  |  |  |  |
| 2.0% | 1,384 | *120* | 145 | 360 | *-62* | 123,311 | *32* | 6,978,508 | *1,011,518* | 31,861 |
| 2.5% | 1,364 | *100* | 129 | 366 | *-56* | 123,312 | *33* | 6,886,822 | *919,832* | 28,152 |
| 3.0% | 1,349 | *85* | 116 | 370 | *-51* | 123,312 | *33* | 6,810,354 | *843,363* | 25,597 |
| 3.5% | 1,336 | *72* | 104 | 375 | *-47* | 123,312 | *33* | 6,745,561 | *778,570* | 23,745 |
| 4.0% | 1,325 | *61* | 94 | 378 | *-44* | 123,312 | *32* | 6,689,988 | *722,997* | 22,358 |
| 4.5% | 1,316 | *52* | 86 | 382 | *-40* | 123,311 | *32* | 6,641,864 | *674,873* | 21,296 |
| 5.0% | 1,309 | *45* | 78 | 385 | *-37* | 123,310 | *31* | 6,599,868 | *632,877* | 20,471 |
| 5.5% | 1,302 | *38* | 71 | 387 | *-35* | 123,310 | *30* | 6,562,985 | *595,995* | 19,828 |
| 6.0% | 1,296 | *32* | 65 | 390 | *-32* | 123,309 | *29* | 6,530,418 | *563,427* | 19,327 |
| 6.5% | 1,291 | *27* | 60 | 392 | *-30* | 123,308 | *28* | 6,501,526 | *534,536* | 18,941 |
| 7.0% | 1,287 | *23* | 55 | 394 | *-28* | 123,307 | *27* | 6,475,791 | *508,800* | 18,650 |
| 7.5% | 1,283 | *19* | 51 | 396 | *-26* | 123,306 | *26* | 6,452,783 | *485,792* | 18,440 |
| 8.0% | 1,280 | *16* | 47 | 397 | *-24* | 123,305 | *25* | 6,432,146 | *465,155* | 18,298 |
| 8.5% | 1,277 | *13* | 43 | 399 | *-23* | 123,304 | *25* | 6,413,580 | *446,589* | 18,217 |
| 9.0% | 1,275 | *11* | 40 | 400 | *-22* | 123,303 | *24* | 6,396,833 | *429,842* | 18,190 |
| 9.5% | 1,272 | *8* | 37 | 402 | *-20* | 123,302 | *23* | 6,381,688 | *414,697* | 18,211 |
| 10.0% | 1,270 | *7* | 35 | 403 | *-19* | 123,301 | *22* | 6,367,959 | *400,969* | 18,275 |

Cohorts of 8.27 million men aged 45 to 69 were followed to age 90. Results are based on 10,000 simulations.

# Table E: Outcomes per 10,000 men of age-based and precision screening compared with no screening from age 50

| **Screening strategy** | **Prostate cancer cases (n)** | ***Difference with no screening*** | **Overdiagnosed cases (n)** | **Deaths from prostate cancer (n)** | ***Difference with no screening*** | **QALYs (n)** | ***Difference with no screening*** | **Costs (£)** | ***Difference with no screening*** | **ICER (£ / QALY gained)** |
| --- | --- | --- | --- | --- | --- | --- | --- | --- | --- | --- |
| No screening | 1,241 |  | - | 426 |  | 113,994 |  | 6,331,963 |  |  |
| Age-based screening | 1,485 | *244* | 223 | 339 | *-87* | 114,018 | *24* | 7,432,457 | *1,100,494* | 45,218 |
| Precision screening (10-year AR) |  |  |  |  |  |  |  |  |  |  |
| 2.0% | 1,398 | *157* | 175 | 352 | *-73* | 114,031 | *37* | 7,530,173 | *1,198,209* | 32,502 |
| 2.5% | 1,376 | *135* | 159 | 357 | *-68* | 114,032 | *39* | 7,430,833 | *1,098,870* | 28,501 |
| 3.0% | 1,356 | *116* | 144 | 362 | *-63* | 114,033 | *39* | 7,342,796 | *1,010,832* | 25,616 |
| 3.5% | 1,340 | *99* | 131 | 367 | *-59* | 114,034 | *40* | 7,265,233 | *933,269* | 23,465 |
| 4.0% | 1,325 | *85* | 120 | 371 | *-55* | 114,033 | *40* | 7,196,955 | *864,991* | 21,819 |
| 4.5% | 1,313 | *72* | 109 | 375 | *-51* | 114,033 | *39* | 7,136,766 | *804,803* | 20,533 |
| 5.0% | 1,303 | *62* | 100 | 378 | *-47* | 114,032 | *39* | 7,083,581 | *751,618* | 19,514 |
| 5.5% | 1,293 | *53* | 91 | 382 | *-44* | 114,031 | *38* | 7,036,452 | *704,489* | 18,697 |
| 6.0% | 1,286 | *45* | 84 | 385 | *-41* | 114,030 | *37* | 6,994,568 | *662,604* | 18,040 |
| 6.5% | 1,279 | *38* | 77 | 387 | *-38* | 114,029 | *36* | 6,957,234 | *625,270* | 17,510 |
| 7.0% | 1,273 | *32* | 71 | 390 | *-36* | 114,028 | *35* | 6,923,862 | *591,898* | 17,085 |
| 7.5% | 1,268 | *27* | 66 | 392 | *-34* | 114,027 | *34* | 6,893,949 | *561,986* | 16,746 |
| 8.0% | 1,263 | *22* | 61 | 394 | *-31* | 114,026 | *32* | 6,867,068 | *535,104* | 16,481 |
| 8.5% | 1,259 | *18* | 56 | 396 | *-30* | 114,025 | *31* | 6,842,850 | *510,886* | 16,280 |
| 9.0% | 1,256 | *15* | 52 | 398 | *-28* | 114,024 | *30* | 6,820,980 | *489,017* | 16,134 |
| 9.5% | 1,253 | *12* | 48 | 399 | *-26* | 114,023 | *29* | 6,801,188 | *469,224* | 16,037 |
| 10.0% | 1,250 | *9* | 45 | 401 | *-25* | 114,022 | *28* | 6,783,237 | *451,274* | 15,984 |

Cohorts of 6.34 million men aged 50 to 69 were followed to age 90. Results are based on 10,000 simulations.

# Table F: Outcomes per 10,000 men of age-based and precision screening compared with no screening from age 60

| **Screening strategy** | **Prostate cancer cases (n)** | ***Difference with no screening*** | **Overdiagnosed cases (n)** | **Deaths from prostate cancer (n)** | ***Difference with no screening*** | **QALYs (n)** | ***Difference with no screening*** | **Costs (£)** | ***Difference with no screening*** | **ICER (£ / QALY gained)** |
| --- | --- | --- | --- | --- | --- | --- | --- | --- | --- | --- |
| No screening | 1,200 |  | - | 430 |  | 104,117 |  | 6,637,163 |  |  |
| Age-based screening | 1,345 | *146* | 123 | 373 | *-57* | 104,149 | *32* | 7,406,417 | *769,254* | 23,931 |
| Precision screening (10-year AR) |  |  |  |  |  |  |  |  |  |  |
| 2.0% | 1,329 | *129* | 116 | 375 | *-55* | 104,151 | *35* | 7,588,593 | *951,430* | 27,501 |
| 2.5% | 1,320 | *120* | 112 | 376 | *-53* | 104,152 | *35* | 7,556,633 | *919,470* | 26,161 |
| 3.0% | 1,311 | *111* | 107 | 378 | *-51* | 104,152 | *35* | 7,521,723 | *884,559* | 24,955 |
| 3.5% | 1,302 | *102* | 102 | 380 | *-49* | 104,152 | *36* | 7,485,597 | *848,433* | 23,890 |
| 4.0% | 1,293 | *93* | 96 | 382 | *-47* | 104,152 | *35* | 7,449,458 | *812,294* | 22,956 |
| 4.5% | 1,284 | *85* | 91 | 385 | *-45* | 104,152 | *35* | 7,414,102 | *776,938* | 22,140 |
| 5.0% | 1,276 | *76* | 86 | 387 | *-43* | 104,152 | *35* | 7,380,033 | *742,870* | 21,427 |
| 5.5% | 1,269 | *69* | 81 | 389 | *-41* | 104,151 | *34* | 7,347,552 | *710,388* | 20,805 |
| 6.0% | 1,262 | *62* | 76 | 391 | *-39* | 104,150 | *34* | 7,316,815 | *679,652* | 20,263 |
| 6.5% | 1,255 | *55* | 71 | 393 | *-37* | 104,150 | *33* | 7,287,887 | *650,724* | 19,791 |
| 7.0% | 1,249 | *50* | 67 | 395 | *-35* | 104,149 | *32* | 7,260,766 | *623,603* | 19,383 |
| 7.5% | 1,244 | *44* | 63 | 396 | *-33* | 104,148 | *31* | 7,235,410 | *598,247* | 19,032 |
| 8.0% | 1,239 | *39* | 59 | 398 | *-32* | 104,148 | *31* | 7,211,751 | *574,588* | 18,731 |
| 8.5% | 1,234 | *35* | 56 | 400 | *-30* | 104,147 | *30* | 7,189,706 | *552,543* | 18,477 |
| 9.0% | 1,230 | *31* | 52 | 401 | *-28* | 104,146 | *29* | 7,169,183 | *532,020* | 18,265 |
| 9.5% | 1,226 | *27* | 49 | 403 | *-27* | 104,145 | *28* | 7,150,088 | *512,925* | 18,093 |
| 10.0% | 1,223 | *23* | 46 | 404 | *-26* | 104,144 | *28* | 7,132,327 | *495,164* | 17,956 |

Cohorts of 2.88 million men aged 60 to 69 were followed to age 90. Results are based on 10,000 simulations. Note that the different sensitivity analyses running from different starting ages are not directly comparable due to different numbers of men, and different lengths of follow-up impacting discounting.

# Appendix references

1. Curtis L, Burns A. Unit Costs of Health and Social Care 2016 [Internet]. Available: https://www.pssru.ac.uk/pub/uc/uc2016/full.pdf?uc=2016-full

2. Department of Health. NHS reference costs 2015 to 2016 [Internet]. [cited 19 Jul 2018]. Available: https://www.gov.uk/government/publications/nhs-reference-costs-2015-to-2016

3. The National Prostate Cancer Audit Annual Report 2017 [Internet]. London; 2017. Available: www.npca.org.uk

4. NICE. Costing statement: prostate cancer: diagnosis and treatment [Internet]. London; 2014. Available: https://www.nice.org.uk/guidance/cg179/resources/costing-statement-248688109

5. Surgeons TBA of U. Analyses of Radical Prostatectomies performed between January 1st and December 31st 2016. London, U.K.; 2016.

6. National Collaborating Centre for Cancer. Prostate Cancer: Diagnosis and Treatment: Clinical Guideline [Internet]. London; 2014. Available: https://www.nice.org.uk/guidance/CG175

7. Ball C, Temple-Murray A, Fell K, Crellin A. Dose Fractionation of Radical Radiotherapy to Prostate Cancer in the UK [Internet]. Available: http://www.natcansat.nhs.uk/rt/pubs.aspx?s=2

8. Faculty of Clinical Oncology of The Royal College of Radiologists. Radiotherapy dose fractionation, second edition [Internet]. 2016. Available: https://www.rcr.ac.uk/system/files/publication/field_publication_files/bfco163_dose_fractionation_2nd_ed_march2017.pdf

9. NICE. Docetaxel for the treatment of hormone-refractory metastatic prostate cancer. Technology appraisal (TA101) [Internet]. London; 2006. Available: https://www.nice.org.uk/guidance/ta101/chapter/4-Evidence-and-interpretation

10. Round J, Jones L, Morris S. Estimating the cost of caring for people with cancer at the end of life: A modelling study. Palliat Med. 2015;29: 899–907. doi:10.1177/0269216315595203

11. NICE. Prostate cancer: NICE Pathway [Internet]. [cited 20 Jul 2018]. Available: https://pathways.nice.org.uk/pathways/prostate-cancer#path=view%3A/pathways/prostate-cancer/prostate-cancer-overview.xml&content=view-index

12. Fenton JJ, Weyrich MS, Durbin S, Liu Y, Bang H, Melnikow J. Prostate-Specific Antigen–Based Screening for Prostate Cancer. Jama. 2018;95817. doi:10.1001/jama.2018.3712

13. Rosario DJ, Lane JA, Metcalfe C, Donovan JL, Doble A, Goodwin L, et al. Short term outcomes of prostate biopsy in men tested for cancer by prostate specific antigen: prospective evaluation within ProtecT study. BMJ. 2012;344: d7894–d7894. doi:10.1136/bmj.d7894

14. Hamdy FC, Donovan JL, Lane JA, Mason M, Metcalfe C, Holding P, et al. 10-Year Outcomes after Monitoring, Surgery, or Radiotherapy for Localized Prostate Cancer. N Engl J Med. 2016;375: 1415–1424. doi:10.1056/NEJMoa1606220

15. Public Health England & Cancer Research UK. Chemotherapy, Radiotherapy and Surgical Tumour Resections in England [Internet]. [cited 19 Jul 2018]. Available: https://www.cancerdata.nhs.uk/treatments

16. Dahabreh IJ, Chung M, Balk EM, Yu WW, Mathew P. Annals of Internal Medicine Active Surveillance in Men With Localized Prostate Cancer. 2011;

17. Lane JA, Donovan JL, Davis M, Walsh E, Dedman D, Down L, et al. Active monitoring, radical prostatectomy, or radiotherapy for localised prostate cancer: study design and diagnostic and baseline results of the ProtecT randomised phase 3 trial. Lancet Oncol. 2014;15: 1109–1118. doi:10.1016/s1470-2045(14)70361-4

18. Donovan JL, Hamdy FC, Lane JA, Mason M, Metcalfe C, Walsh E, et al. Patient-Reported Outcomes after Monitoring, Surgery, or Radiotherapy for Prostate Cancer. N Engl J Med. 2016;375: 1425–1437. doi:10.1056/NEJMoa1606221

19. Ahmed HU, El-Shater Bosaily A, Brown LC, Gabe R, Kaplan R, Parmar MK, et al. Diagnostic accuracy of multi-parametric MRI and TRUS biopsy in prostate cancer (PROMIS): a paired validating confirmatory study. Lancet. 2017;389: 815–822. doi:10.1016/S0140-6736(16)32401-1

20. Schröder FH, Hugosson J, Roobol MJ, Tammela TLJ, Zappa M, Nelen V, et al. Screening and prostate cancer mortality: Results of the European Randomised Study of Screening for Prostate Cancer (ERSPC) at 13 years of follow-up. The Lancet. 2014. doi:10.1016/S0140-6736(14)60525-0

21. Ara R, Brazier JE. Using health state utility values from the general population to approximate baselines in decision analytic models when condition-specific data are not available. Value Heal. 2011;14: 539–545. doi:10.1016/j.jval.2010.10.029

22. Pharoah PDP, Sewell B, Fitzsimmons D, Bennett HS, Pashayan N. Appendix: Cost effectiveness of the NHS breast screening programme: Life table model. BMJ. 2013;346: 1–12. doi:10.1136/bmj.f2618

23. Downing A, Wright P, Hounsome L, Selby P, Wilding S, Watson E, et al. Quality of life in men living with advanced and localised prostate cancer in the UK: a population-based study. Lancet Oncol. 2019;2045. doi:10.1016/S1470-2045(18)30780-0

24. Heijnsdijk EAM, Wever EM, Auvinen A, Hugosson J, Ciatto S, Nelen V, et al. Quality-of-Life Effects of Prostate-Specific Antigen Screening. N Engl J Med. 2012;367: 595–605. doi:10.1056/NEJMoa1201637

25. Pharoah PDP, Sewell B, Fitzsimmons D, Bennett HS, Pashayan N. Cost effectiveness of the NHS breast screening programme: Life table model. BMJ. 2013;346: 1–8. doi:10.1136/bmj.f2618

26. Cancer Research UK. Prostate cancer survival statistics [Internet]. [cited 30 Jul 2018]. Available: https://www.cancerresearchuk.org/health-professional/cancer-statistics/statistics-by-cancer-type/prostate-cancer/survival#heading-Zero

27. Heijnsdijk EA, Wever EM, Auvinen A, Hugosson J, Ciatto S, Nelen V, et al. Quality-of-life effects of prostate-specific antigen screening. N Engl J Med. 2012;367: 595–605. doi:10.1056/NEJMoa1201637

28. Venderbos LDF, Aluwini S, Roobol MJ, Bokhorst LP, Oomens EHGM, Bangma CH, et al. Long-term follow-up after active surveillance or curative treatment: quality-of-life outcomes of men with low-risk prostate cancer. Qual Life Res. 2017;26: 1635–1645. doi:10.1007/s11136-017-1507-7

29. Lardas M, Liew M, van den Bergh RC, De Santis M, Bellmunt J, Van den Broeck T, et al. Quality of Life Outcomes after Primary Treatment for Clinically Localised Prostate Cancer: A Systematic Review. Eur Urol. 2017;72: 869–885. doi:10.1016/j.eururo.2017.06.035

30. Korfage IJ, Essink-Bot M-L, Borsboom GJJM, Madalinska JB, Kirkels WJ, Habbema JDF, et al. Five-year follow-up of health-related quality of life after primary treatment of localized prostate cancer. Int J Cancer. 2005;116: 291–296. doi:10.1002/ijc.21043

31. Pashayan N, Duffy SW, Pharoah P, Greenberg D, Donovan J, Martin RM, et al. Mean sojourn time, overdiagnosis, and reduction in advanced stage prostate cancer due to screening with PSA: Implications of sojourn time on screening. Br J Cancer. 2009;100: 1198–1204. doi:10.1038/sj.bjc.6604973

32. Fenton JJ, Weyrich MS, Durbin S, Liu Y, Bang H, Melnikow J. Prostate-Specific Antigen–Based Screening for Prostate Cancer: A Systematic Evidence Review for the U.S. Preventive Services Task Force. Evidence Synthesis No. 154. AHRQ Publication No. 17-05229-EF-1. Rockville, MD; 2018.

33. Office for National Statistics. Cancer Registration Statistics, England [Internet]. [cited 18 Jun 2018]. Available: https://www.ons.gov.uk/peoplepopulationandcommunity/healthandsocialcare/conditionsanddiseases/datasets/cancerregistrationstatisticscancerregistrationstatisticsengland

34. Office for National Statistics. Death Registrations Summary Statistics, England and Wales 2016 [Internet]. Newport, Wales; 2017. Available: https://www.ons.gov.uk/peoplepopulationandcommunity/birthsdeathsandmarriages/deaths/datasets/deathregistrationssummarytablesenglandandwalesreferencetables

35. Briggs ADM, Wolstenholme J, Blakely T, Scarborough P. Choosing an epidemiological model structure for the economic evaluation of non-communicable disease public health interventions. Popul Health Metr. 2016;14: 17. doi:10.1186/s12963-016-0085-1

36. Office for National Statistics. Population estimates [Internet]. [cited 17 Jul 2018]. Available: https://www.nomisweb.co.uk/query/construct/components/simpleapicomponent.aspx?menuopt=20002&subcomp=#

37. Office for National Statistics. Mortality statistics [Internet]. [cited 15 Aug 2018]. Available: https://www.nomisweb.co.uk/

38. Office for National Statistics. Population estimates [Internet]. [cited 19 Jun 2018]. Available: https://www.ons.gov.uk/peoplepopulationandcommunity/populationandmigration/populationestimates/datasets/populationestimatesrevisiontool

39. Draisma G, Etzioni R, Tsodikov A, Mariotto A, Wever E, Gulati R, et al. Lead time and overdiagnosis in prostate-specific antigen screening: Importance of methods and context. J Natl Cancer Inst. 2009;101: 374–383. doi:10.1093/jnci/djp001

40. Young GJ, Harrison S, Turner EL, Walsh EI, Oliver SE, Ben-Shlomo Y, et al. Prostate-specific antigen (PSA) testing of men in UK general practice: a 10-year longitudinal cohort study. BMJ Open. 2017;7: e017729. doi:10.1136/bmjopen-2017-017729

41. Dadaev T, Saunders EJ, Newcombe PJ, Anokian E, Leongamornlert DA, Brook MN, et al. Fine-mapping of prostate cancer susceptibility loci in a large meta-analysis identifies candidate causal variants. Nat Commun. 2018;9: 2256. doi:10.1038/s41467-018-04109-8

42. Schumacher FR, Al Olama AA, Berndt SI, Benlloch S, Ahmed M, Saunders EJ, et al. Association analyses of more than 140,000 men identify 63 new prostate cancer susceptibility loci. Nat Genet. 2018;50: 928–936. doi:10.1038/s41588-018-0142-8

43. DevCan [Internet]. Bethesda, M.D.: US NIH National Cancer Institute; Available: https://surveillance.cancer.gov/devcan/download

44. NICE. Guide to the methods of technology appraisal 2013 [Internet]. London: National Institute for Health and Care Excellence; 2013. Available: https://www.nice.org.uk/process/pmg9/resources/guide-to-the-methods-of-technology-appraisal-2013-pdf-2007975843781
