# Supplementary material for: Polygenic risk-tailored screening for prostate cancer: A benefit–harm and cost-effectiveness modelling study
Source: PLoS Med. 2019 Dec 20;16(12):e1002998. doi: 10.1371/journal.pmed.1002998 (PMC6924639; doi:10.1371/journal.pmed.1002998)
Supplement: Attachment — Submitted filename: Response to reviewers letter.docx [file pmed.1002998.s003.docx]

Dear Dr. Misra,

Thank you very much for the opportunity to revise our manuscript and for the helpful feedback and comments.

-----------------------------------------------------------

Requests from the editors:

Abstract-please explain the life table model briefly

The first line of the methods now reads:

A life-table model, projecting age-specific prostate cancer incidence and mortality, was developed of a hypothetical cohort of 4.48 million men in England aged 55 to 69 years with follow-up to age 90.

Abstract-please clarify what the analysis from the health service perspective would entail. If this is intention to pay or cost burden, please include this information.

We have added to the relevant sentence in the abstract, so that it now reads:

The analysis was undertaken from the health service perspective, including direct costs borne by the health system for risk assessment, screening, diagnosis, and treatment.

Abstract-in the methods/findings section please include a sentence about the limitations of your methodology

We have included the following sentence to the last end of paragraph on methods:

Our model has some limitation related to its cohort-based structure.

Abstract-please quantify all results using p-values and 95% confidence intervals

Reflecting the probabilistic nature of the presented model results, we have added 95% uncertainty intervals as we do not have statistical inference to calculate p-values and 95% confidence intervals.

Abstract-in the conclusions section please clarify that these are based on a modelling analysis

We have now adjusted the conclusion to clarify both that it is based on a modelling analysis, and in response to the next comment regarding clinical application, so that it now reads:

Based on the results of this modelling study, offering screening to men at higher risk could potentially reduce overdiagnosis, improve the benefit-harm trade-off, and the cost-effectiveness of a prostate cancer screening program.

Abstract-please tone down the language in the abstract to reflect that there is no clinical application of polygenic risk scores in prostate cancer screening

We have adjusted both our background and conclusion to avoid suggesting there is any current clinical application of polygenic risk scores in prostate cancer screening:

**Background**

The US Preventive Services Task Force supports individualized decision making for PSA-based screening in men aged 55-69. Knowing how the potential benefits and harms of screening vary by an individual’s risk of developing prostate cancer could inform decision-making about screening at both an individual and population level…

**Conclusions**

Based on the results of this modelling study, offering screening to men at higher risk could potentially reduce overdiagnosis, improve the benefit-harm trade-off, and the cost-effectiveness of a prostate cancer screening program.

Author summary- At this stage, we ask that you include a short, non-technical Author Summary of your research to make findings accessible to a wide audience that includes both scientists and non-scientists. The Author Summary should immediately follow the Abstract in your revised manuscript. This text is subject to editorial change and should be distinct from the scientific abstract. Please see our author guidelines for more information: <https://journals.plos.org/plosmedicine/s/revising-your-manuscript#loc-author-summary>

We have added an Author Summary following the Abstract in the manuscript (page 3):

**Author Summary**

**Why Was This Study Done?**

- Prostate cancer screening using prostate-specific antigen has been shown to lead to a reduction in prostate cancer-specific mortality, at the expense of overdiagnosis and overtreatment.
- Genome wide association studies have identified more than 160 common genetic variants that, when combined together as a polygenic risk score, can be used to develop a tailored screening programme for prostate cancer.
- The proportion of men overdiagnosed has been shown to vary by polygenic risk, therefore a risk-tailored screening based on age and polygenic risk profile may improve the balance of benefits and harms of a screening programme for prostate cancer.

**What Did the Researchers Do and Find?**

- We developed a mathematical model that simulated hypothetical cohorts of 4.48 million men aged 55 to 69 in England.
- Using this model, we analysed the balance of benefits and harms in terms of prostate cancer-specific mortality reduction against overdiagnosis, as well as the cost-effectiveness, of the introduction of a risk-tailored screening programme for prostate cancer based on age and polygenic risk.
- We compared risk-tailored screening to age-based screening and no screening.

**What Do These Findings Mean?**

- Based on this model, we show that a polygenic risk-tailored screening programme would reduce overdiagnosis, maintain the mortality benefits of age-based screening, and improve the cost-effectiveness of a screening programme for prostate cancer.
- The ideal threshold for risk-tailored screening will depend on societal judgement of the trade-off between the benefits and harms of screening.
- Future prospective evaluations are needed to verify these findings**.**

References-please use square brackets and remove italics from citations. Please use the "Vancouver" style for reference formatting, and see our website for other reference guidelines <https://journals.plos.org/plosmedicine/s/submission-guidelines#loc-references>

Now adjusted – we apologise for not having done this before the initial submission.

Introduction-Please include a sentence about PSA testing, why there is a substantial risk of false positive diagnosis. The second sentence requires clarification, please revise or consider removing.

We have expanded the first sentence of the Introduction to add the risk of false positives alongside overdiagnosis. We have revised and clarified the second sentence which explains what we mean by overdiagnosed cancers.

Screening with prostate-specific antigen (PSA) test could reduce death from prostate cancer in some men, but at the cost of substantial numbers overdiagnosed, as well as false positive results.[1] Overdiagnosed cancers are the screen-detected cancers that would not otherwise have come to light during an individual’s lifetime,[2] whose diagnosis and related treatment can lead to avoidable physical and psychological harms whilst also incurring an economic cost.[3]

Introduction-please introduce polygenic risk at first view and explain how this may enable tailored screening

We’ve added to the second paragraph of the Introduction to describe polygenic risks further and why this may enable tailored screening, and added two figures (S1 Figure 3 and 4) to the Appendix to illustrate the relationship between the proportion of all cancers amongst different percentiles of the risk distribution:

Genome-wide association studies have identified common susceptibility loci that together explain approximately 37% of the familial relative risk of prostate cancer.[4] Individually, these loci have little clinical significance.[5] However, together they define a risk distribution with a variance of 0.68 and AUC of 0.72 that can be used to stratify individuals into groups at higher and lower risk of developing prostate cancer,[2,6] enabling tailored screening by risk group. Individuals in the 1st and 99^th^ percentiles of the polygenic risk distribution have relative risks of developing prostate cancer of 0.09 and 5.52, respectively, compared to the population mean. Almost 49% of prostate cancers occur in those men in the highest 20% of the polygenic risk distribution, and only 7% amongst those in the lowest 20% of the risk distribution (S1 Figure 3). Avoiding screening of those at lower risk may consequently reduce the harms of screening without a commensurate loss of benefit. The proportion of prostate cancers overdiagnosed varies inversely with polygenic risk, with almost 50% lower overdiagnosis in men in the highest quartile compared with lowest quartile of polygenic risk.[2] This approach to screening would require genotyping of all men for the purpose of risk assessment. However, the additional costs of population-wide genotyping may be offset by lower levels of overtreatment.

Methods-Please ensure that the correct link to the Python code has been provided as the current link is not working

Our apologies, the hyperlink now works.

Methods-Please provide paragraph or line numbers in the CHEERS checklist as page numbers are likely to change

We’ve updated our CHEERS checklist to use paragraph numbers.

Methods-Please briefly explain the life table model

We have added to the first paragraph of the Methods sub-section ‘Model Structure’ so that it now reads:

We developed a life-table model (S1 Figure 1) to simulate cohorts of men under three scenarios: no screening; age-based screening with PSA; and risk-tailored screening (henceforth, precision screening). Our life-table is a cohort-based Markov model that estimates the age-specific incidence of prostate cancer, deaths from prostate cancer and from other causes.

Discussion- please include something like “according to our modelling analysis...” at the start of the section

The first line of the discussion now reads:

This modelling analysis has shown that precision screening based on age and polygenic risk would reduce overdiagnosis whilst preserving most of the mortality benefits of age-based screening for prostate cancer.

Last sentence of the conclusion- please revise to clarify that a RCT would be essential, instead of valuable

We completely agree that prospective evaluation is essential before precision screening could be advocated as a policy position, and have altered the last sentence as follows:

These findings require verification by a prospective randomized evaluation.

Overall please use careful language to indicate that the results are based on a model

Comments from the reviewers:

Reviewer #1: In this article, the authors use a life-table based model to estimate the benefits, harms and cost-effectiveness of a program that screens men in the U.K. for prostate cancer based on their risk (based on age and polygenic profile) versus age-based screening and no screening.

The authors found that risk-based screening using a risk threshold for screening commencement of 5% or over was cost-effective (using a willingness-to-pay threshold of £20,000 per QALY) when compared with age-based screening.

Overall this is an interesting study that is addressing the important question of how to optimize prostate cancer screening and limit the harms associated with the high proportion of overdiagnosed cases. However I believe that the modelling approach used in this paper is not the most appropriate and restricts the possible characteristics of a risk-based screening program that can be explored and evaluated.

We appreciate the positive comments about the importance of the question and the interest of the study. We very much agree on the importance of attempting to limit the harms of prostate cancer screening.

For example, risk-based screening programs using different screening intervals and/or PSA thresholds cannot be evaluated because the relative risk of prostate cancer mortality in the presence of screening is taken directly from the ERSPC trial and therefore the model in this manuscript is constrained to do what was done in the trial.

We have chosen to base relevant model parameters on the ERSPC trial as we felt this was the most appropriate evidence to underpin our methods. There have been several major trials of prostate cancer screening with PSA, including ERSPC, PLCO, and CAP, as well as a Cochrane review and meta-analysis. One-off screening with PSA was not supported by the findings of CAP (doi:10.1001/jama.2018.0154). ERSPC and PLCO came to differing conclusions about the benefits of PSA screening approximately every four years; however, Tsodikov and colleagues found (doi:10.7326/M16-2586) that, once differences in implementation and setting were accounted for, both studies provide similar evidence of prostate cancer mortality reductions with screening. As the Cochrane review does not take this into account, we chose to base our findings on the ERSPC. This approach is also consistent with that taken by the Fred Hutchinson Cancer Research Center’s CISNET model which was validated against the prostate cancer mortality from ERSPC (<https://doi.org/10.1371/journal.pone.0211918>).

We agree that it would be valuable to understand the impact of varying screening intervals or using alternative PSA thresholds. Our model does have the flexibility to tackle this, however there are few consistent empirical data on which to base changes to the screening interval or PSA threshold for age-based screening, and none in the context of polygenic risk. We therefore kept to the thresholds and screening intervals of the core group of the ERSPC as the best current evidence, and have noted this in the discussion:

In a precision screening programme, in addition to altering the screening start age, the frequency of screening could be varied according to risk. For example, men at higher risk may receive more frequent screening and men at lower risk receive less frequent or no screening. Intensified screening could improve the benefit-harm trade-offs if the sojourn time – the time it takes to progress from preclinical screen-detectable cancer to clinically detectable cancer – varies with risk-level.[36] In the absence of these data in the context of polygenic risk, the impact of varying screening intervals was not estimated.

Well calibrated and validated microsimulation models that use a more detailed natural history of prostate cancer are a better alternative to perform a comprehensive evaluation of a risk-based screening program for prostate cancer. Such models have been developed for some countries (e.g. the United States and the Netherlands - see <https://cisnet.cancer.gov/prostate/>) and have been successfully used in several evaluations, some of which are cited in the manuscript. This alternative approach would for example

*	Allow different screening intervals, PSA thresholds, starting/stopping ages for screening to be evaluated

*	Evaluate emerging triage technologies (i.e. not all men with PSA > 3ng/ml would be sent directly to biopsy - this could potentially further reduce overdiagnosis);

*	Directly assert from the simulation whether a prostate cancer case is an overdiagnosed case as opposed to using a separate model for this classification;

*	Better model the introduction of screening and the transitory effects that could occur.

A microsimulation model of prostate cancer that accounted for individual risk profile would be an interesting and worthwhile addition to the current status of prostate cancer modelling research.

..

The authors rightly state in the appendix that a life table based model is simpler and limits the number of unknown parameters when compared with microsimulation models. However there are various methods that are regularly used to parametrize microsimulation models together with uncertainty analysis methods that look at how sensitive the outcomes are to the fitted parameter sets.

We agree that microsimulation is the most flexible method to analyse screening programmes but has several disadvantages of importance to this study which led us to choose to take a life-table approach. At present we have no data on which to base a detailed natural history of prostate cancer in the context of polygenic risk. To take advantage of microsimulation in a UK or US context, we would benefit from data on how sojourn time and prostate cancer incidence in different ethnicities varies by risk, which are not currently available. Therefore, any microsimulation model of polygenic risk in prostate cancer would necessarily be relatively simplistic, minimising the advantages of modelling individual variation. Nevertheless, given the relative opacity of microsimulation models, the transparency of the analysis would be diminished. As relevant data become available, microsimulation will likely be a future step.

By taking a life-table approach, and publishing the code alongside the paper, we have aimed for transparency, and have taken account of the wide range of likely individual response to polygenic screening with probabilistic analyses. We present sensitivity analyses of different starting ages for screening in the Appendix (S1 Tables 4-6). As noted in the discussion, our model compares favourably to comparable CISNET microsimulations, attesting to its robustness:

There have been no comparable studies estimating the impact of polygenic risk-tailored screening in prostate cancer. However, the conclusions reached from our precision and age-based screening models compare favourably with a micro-simulation model from the US[37], attesting to the underlying robustness of our model in spite of the differences in model design and assumptions. Using a microsimulation model and a selective treatment strategy involving initial conservative management for those with localised cancer (Gleason score <7 and stage T2a), Roth and colleagues state that quadrennial age-based screening between the ages of 55 and 69 with a PSA cut-off of 3ng/ml would lead to 30 additional QALYs per 10,000 men screened (37 in this analysis).[37]

Lastly, I tried to access the Python code in <https://github.com/callta/precision_screening_pca> but the repository was empty (tried to access this on 2019-08-30).

Our apologies, this has now been fixed.

Reviewer #2: Callender et al provide a timely and interesting comparative effectiveness analysis of prostate cancer screening. They show convincingly that a precision approach offers substantial benefits in terms of reduced costs per QALY and reduced over-diagnosis, at given thresholds of lives lost to prostate cancer. They argue that risk-based screening based instead of on age, on combined genetics and age-adjusted 10-year risk of 4% to 7% provides the optimal balance that is likely to meet willingness-to-pay recommendations for the UK.

Thank you for your positive comments.

There is however one important element of the study that is missing, and that is any evaluation of the performance of polygenic risk assessment. The main reference [7] given for justifying the parameter they include in their modeling is a 2002 paper on breast cancer, and the only other reference to a PRS is [28] in the last sentence of the manuscript. In addition to that study, a quick search identified two more (PMID: 30366021, PMID: 29892016) with polygenic scores incorporating from 7 to 147 SNPs, and it should now be possible based on the data in the latter study to generate a truly genome-wide score based on millions of variants. I think it is essential to consider in this paper the impact of the likely improvement in diagnostic utility of PRS as they explain more of the (genetic) variance of disease. Relative risks in the top percentiles are now approaching 3X greater than the median; and similarly at the low end of the scale it should be possible to identify individuals whose negative predictive value is such that it provides even more utility than positive predictive value. Presumably a composite score combining age, polygenic risk, and other prostate cancer risk factors (eg family history, prior PSA tests), and ancestry will do even better.

Thank you for noticing that only reference 7 is given. We have now changed the relevant paragraph to reference the source we used to derive our risk distribution: Dadaev and colleagues’ fine-mapping study (DOI: 10.1038/s41467-018-04109-8), which extends on the work of Schumacher and colleagues (PMID: 29892016) and provides the most up-to-date prostate cancer susceptibility loci:

The varianceof the polygenic risk distribution was calculated as 0.68, based on known prostate cancer susceptibility variants (see S1 Appendix for further details).[6,9]

In place of PRS based on SNPs that have reached genome-wide significance, genome-wide scores based on millions of variants including those that have not reached genome-wide significance are currently thought to only provide modest, at best, improvements in discriminatory power, due to the challenges in distinguishing noise from those SNPs that genuinely confer a risk of disease (http://dx.doi.org/10.1038/nrg.2016.27). As a result, we have based the risk distribution used on those SNPs known to have genome-wide significance.

We agree that the paper will be improve by further discussion of the predictive utility of PRS. To do this, we’ve added to the Introduction to contextualise the predictive utility and discriminatory power of PRS in screening, so that the relevant paragraph now reads:

Genome-wide association studies have identified common susceptibility loci that together explain approximately 37% of the familial relative risk of prostate cancer.[4] Individually, these loci have little clinical significance.[5] However, together they define a risk distribution with a variance of 0.68 and AUC of 0.72 that can be used to stratify individuals into groups at higher and lower risk of developing prostate cancer,[2,6] enabling tailored screening by risk group. Individuals in the 1st and 99^th^ percentiles of the polygenic risk distribution have relative risks of developing prostate cancer of 0.09 and 5.52, respectively, compared to the population mean. Almost 49% of prostate cancers occur in those men in the highest 20% of the polygenic risk distribution, and only 7% amongst those in the lowest 20% of the risk distribution (S1 Figure 3). Avoiding screening of those at lower risk may consequently reduce the harms of screening without a commensurate loss of benefit. The proportion of prostate cancers overdiagnosed varies inversely with polygenic risk, with almost 50% lower overdiagnosis in men in the highest quartile compared with lowest quartile of polygenic risk.[2] This approach to screening would require genotyping of all men for the purpose of risk assessment. However, the additional costs of population-wide genotyping may be offset by lower levels of overtreatment.

We’ve also added two figures (S1 Figure 3 and 4) to the Appendix to further show the 10-year absolute risk of developing prostate cancer for men aged 55 at different percentiles of the risk distribution, and to show the proportion of prostate cancer cases that would occur in men at different percentiles of the risk distribution.

Our study stratifies men on a combination of age and polygenic risk. Although there are relatively few proven risk factors for prostate cancer, we can expect, similar to models such as BOADICEA in breast cancer (https://doi.org/10.1038/s41436-018- 0406-9), that composite scores including family history will lead to information once these data become available. Family history is often incomplete in clinical settings, however, as ~58% of variation between men in prostate cancer risk is considered to be due to genetic profile (doi: 10.1158/1055-9965.EPI-13-0568), there is also scope for further improvements in PRS, and for polygenic risk scores to be tailored by ethnicity, as these data become available.

Several recent reviews have addressed the use of PRS in clinical contexts. This paper is a really nice example of combining such assessments with real-world prediction of utility. It would be even stronger with commentary on the impact of the discriminatory power of the PRS.

Thank you again for your positive comments and for suggesting the addition regarding the discriminatory power of the PRS, described in our response to the preceding paragraph.

Reviewer #3: This is a promising paper but it needs a few major modifications before finer points can even be assessed.

We appreciate that you think this is a promising paper and hope that our answers below aid its assessment.

The first major issue is that it is unclear that the model has necessarily converged. Only 10,000 model runs were used and not all of the line-by-line differences in Table 2 were in the expected directions. As a result, I suspect that this hasn't converged, and that results found may be slightly different were a large number of have been used and convergence achieved. (If I have misunderstood and this is the authors' expected directions, greater clarity over expectations would be helpful.)

We apologise that the expectations were not as clear as we initially thought. From our perspective, the line-by-line differences in Table 2 are in the expected direction. By comparison with no screening, we expected age-based screening to lead to the greatest number of prostate cancer cases, but also the fewest deaths. We would expect that precision screening may lead to more QALYs as fewer men are screened, leading to fewer overdiagnosed cancers and therefore fewer years lived with cancers, but comparable mortality rates to age-based screening. The initial rise in costs with precision screening is expected due to the costs of implementing polygenic testing itself, offset as the threshold rises by the fewer incremental cancers diagnosed relative to no screening.

We have also checked to ensure that, when running our analyses again using a different random number seed, the results are very similar, such that we feel that the model has converged.

The abstract of the paper is very poor in comparison to the elements of the analysis. Given that the paper's results would find that age-based screening is not cost-effective (ICER at 34k per QALY) vs no-screening, the comparison of age-based and precision screening is largely irrelevant. The comparisons against "no screening" in Table 2 are better but this should be incremental.

In our paper we are attempting to weigh-up both the benefit-to-harm profile and the cost-effectiveness of the different screening options. This reflects the fact that the clinical benefits and harms of screening programmes are usually considered independently from cost-effectiveness analyses. Our analyses are also likely to be of interest beyond the UK, where cost-effectiveness may not be given a prominent role in policy making, or where there are different willingness-to-pay thresholds.

As there is no systematic prostate cancer screening in the UK, we use no screening as our baseline. We then modelled age-based screening as this has been studied in several randomised trials and provides a benchmark for readers when interpreting precision screening. Although we do not find age-based screening to be cost-effective, it prevents the most deaths. This then provides a benchmark comparator, alongside no screening, for us to describe the advantages and disadvantages of precision screening, both in terms of its benefit-to-harm profile and cost-effectiveness. Given that implementation of screening programmes is based on a judgement of acceptable trade-offs in terms of both clinical benefits, harms, and cost-effectiveness, we feel that presenting no screening, age-based and precision screening results in the Abstract will be of interest to readers.

We have re-structured our abstract results in order to make our approach clearer:

Compared to no screening over 35 years follow-up, age-based screening prevented the most deaths from prostate cancer (39,272, 95% uncertainty interval [UI]: 16,792 – 59,685), at the expense of 94,831 (95% UI: 84,827 – 105,630) overdiagnosed cancers. Age-based screening was the least cost-effective strategy studied. The greatest number of quality-adjusted life-years (QALYs) was generated by risk-based screening at a 10-year absolute risk threshold of 4%. At this threshold, risk-based screening led to one-third fewer overdiagnosed cancers (64,384, 95% UI: 57,382 – 72,050), but averted 6.3% fewer (9,695, 95% UI: 2,853 – 15,851) deaths from prostate cancer, by comparison with age-based screening. Relative to no screening, risk-based screening at a 4% 10-year absolute risk threshold was cost-effective in 48.4% and 57.4% of the simulations at willingness-to-pay thresholds of £20,000 ($26,000) and £30,000 ($39,386) per QALY, respectively. The cost-effectiveness of risk-tailored screening improved as the threshold rose.

**Conclusions**

Based on the results of this modelling study, offering screening to men at higher risk could potentially reduce overdiagnosis, improve the benefit-harm trade-off, and the cost-effectiveness of a prostate cancer screening program. The optimal threshold will depend on societal judgements of the appropriate balance of benefits-harms, and cost-effectiveness.

This lack of a truly incremental analysis is the biggest (and most telling) flaw in the paper. The paper does not identify a clear enough decision problem, start with a true decision problem, or present results that answer a clear decision problem. Table 2 provides really helpful information for decision making but does not do so in a very systematic (or indeed a truly incremental) fashion. If each of these lines were taken to be a separate decision option (as they are) then this would be much stronger --- here, with back of the envelope calculations (and removing the options that appear to be dominated or extended dominated), it looks to me that the most cost-effective option is to provide screening a risk threshold of 10% with a CE threshold of 20k per QALY and at 8% for a CE threshold of 30k per QALY. I can identify these figures by using Table 2 and calculating ICERs ... this is what the paper should present (it does indirectly where NMB is presented but only as an

aside in the discussion, rather than as the results).

QALYs	Incr QALYs	Costs	Incr Costs	ICER

NS	46,682,945	2,975,391,145

10%	46,699,140	16,195	3,216,074,093	240,682,948	14,862

9.50%	46,699,649	509	3,226,784,314	10,710,221	21,042

9.00%	46,700,163	514	3,238,490,220	11,705,906	22,774

8.50%	46,700,677	514	3,251,296,757	12,806,537	24,915

8.00%	46,701,187	510	3,265,319,785	14,023,028	27,496

7.50%	46,701,687	500	3,280,686,485	15,366,700	30,733

7.00%	46,702,172	485	3,297,535,256	16,848,771	34,740

6.50%	46,702,631	459	3,316,014,724	18,479,468	40,260

6.00%	46,703,054	423	3,336,281,231	20,266,507	47,911

5.50%	46,703,427	373	3,358,493,767	22,212,536	59,551

5.00%	46,703,733	306	3,382,804,647	24,310,880	79,447

4.50%	46,703,950	217	3,409,343,088	26,538,441	122,297

4.00%	46,704,054	104	3,438,186,910	28,843,822	277,344

Note that on the results provided, age-based screening is dominated and doesn't appear in a sensible table of overall results (but would appear in a CEAC).

In an ideal world, the paper should be reframed around this type of view, since it's the standard approach for economic evaluations to consider all relevant options together and to do so incrementally. The paper should also consider some additional points between 9.5% and 10%, and between 7.5% and 8%, in order to optimise this targetted figure a little more closely.

Although our main analyses, as described above, are presented so as to provide a reader with the results necessary to weigh-up both the benefits and harms of screening, alongside consideration of costs, QALYs, and ICERs relative to no screening, we do provide full incremental analyses analogous to the calculations you have shown above. We used net monetary benefit (NMB) to rank all screening options, and have presented the NMB of each screening alternative, as well as a CEAC, cost-effectiveness frontier, and cost-effectiveness planes in the Appendix (S1 Figures 8-11). We describe this in the ‘Net Monetary Benefit of Screening Strategies’ sub-section of the results, where we agree that, at willingness-to-pay thresholds of £20,000 and £30,000 per QALY, precision screening at risk thresholds of 10% and 8% are the most cost-effective strategies, respectively.

We modelled 10-year absolute risk thresholds up to an upper boundary of 10% so as to provide readers with an indication of how this impacts both the effectiveness of screening, as well as its practical implementation. For example, at a screening 10-year absolute risk threshold of 10%, only a cumulative total of 12.3% of the men would become eligible for at least one screen. This would be highly unlikely to be implemented in reality, given the practical challenges of running a population-based screening programme with so few eligible men, and the relatively small benefit of such an approach in terms of mortality reduction. However, when taking a purely incremental approach, this is the most cost-effective strategy. This highlights why we have chosen to compare both benefits and harms, and cost-effectiveness, selecting strategies based on a broader range of criteria and then each strategy with no screening, which is the current paradigm. Hence our optimal strategies are not the most cost-effective ones.

Once this is done then this should really be presented on a CEAC and with a cost-effectiveness frontier; both analyses need to appear as they're standard practice - but neither do at present.

Our CEAC and cost-effectiveness frontiers are available as S1 Figure 10 and 11 in the Appendix. We would be quite happy to move these to the main paper should the Editors so wish.

Given all this, the authors suggestion at the beginning of the discussion that a threshold of 4% to 7% is optimal is utterly indefensible on economic grounds when this purports to talk about a willingness to pay of 20k or 30k per QALY. At 4%, their own results suggest that this is cost-effective only if society is willing to pay in excess of 250k per QALY. The authors have, it appears, done a nice piece or work here but don't appear to have correctly used the methods that they state --- this is, however, very easily fixed!

In our discussion, we reach the suggested thresholds of 4-7%, based on a judgement of the relative benefits and harms of screening relative to both no screening and age-based screening. From an economic perspective, these thresholds would be cost-effective were they considered relative to no screening. They represent the point which maximises the QALY gains of screening, whilst considering overdiagnosis, deaths averted, and costs. Our analysis has shown that precision screening may provide benefits that warrant further prospective evaluation. In the presence of such a trial, a selected strategy would be compared to age-based and no screening, as we have done, rather than all possible precision strategies. Consequently, we have restricted the manuscript to comparisons of individual screening strategies with no screening or age-based screening, whilst presenting the incremental analyses in the Appendix.

Reviewer #4: I confine my remarks to statistical aspects of this paper.

These were generally fine, but I do have a couple of suggestions.

Many thanks for your time and review which we’ve used to improve the re-submitted work.

p 5 - Instead of four yearly put "four times a year" or "every four years".

I'm a little confused as to the data. If it was simulated (top of p. 5) then how could people be invited to do something (middle of p. 5)?

We’ve changed four yearly throughout to every four years and have re-worded the relevant phrase as it is true that this modelling study did not invite anyone to do anything:

Age-based screening involved PSA testing every four years between the ages of 55 and 69, reflecting the screening interval used in the core analyses of the European Randomized Study of Screening for Prostate Cancer…From the age of 55 to 69, this cohort of men started quadrennial PSA screening at the age at which they reached a specified risk threshold, which was varied between a 2% and 10% 10-year absolute risk of developing prostate cancer.

Table 1 - I can't comment on the values chosen, but the distributions seem reasonable.

Table 2 - maybe put QALY in millions and costs in thousands?

Agreed - this will make the Table easier to read. We’ve adjusted costs, putting them in millions. We have experimented with different formats for the QALYs but found that if we shorten the numbers in the Table it ceases to be possible to identify the threshold that produces the highest number of QALYs. We feel that it may be valuable for readers to be able to see the details of the difference in QALYs between the thresholds so have left them in full.

Figure 1	a) I am not sure how the bottom two rows mesh with the relative risk rows. It seems like there would be age based screening and no screening rates for each 10 year risk. b) I would make this into a line graph with risk on the x axis, and frequency on the y axis, with lines as needed.

We’ve changed this to a line graph as suggested, and improved the wording on the axes so that it no longer appears that there would be age-based and no screening for each 10-year risk.

Figure 2. Dual axis graphs are not good (see the work of William Cleveland). Either make this into two graphs or show e.g. the ratio of QALY to cost.

We have experimented with several different graphs, including displaying the data as two graphs or as a ratio measure, but find that these prevent us from being able to show the key visual comparison of both QALYs and costs together at different points in the risk distribution in an intuitive way, so have kept the Figure at present.

Peter Flom
